# Reaching Vulnerable Populations through Portable and Mobile Dentistry—Current and Future Opportunities

**DOI:** 10.3390/dj7030075

**Published:** 2019-08-01

**Authors:** Shailee Gupta, Muna Hakim, Dishant Patel, Lauren C. Stow, Katherine Shin, Peggy Timothé, Romesh P. Nalliah

**Affiliations:** 1 St. David’s Foundation, Austin, TX 78701, USA; 2 School of Dentistry, University of Michigan, Ann Arbor, MI 48109, USA; 3 Dental Program, University of Adelaide, Adelaide 5005, South Australia; 4 Academic Affairs and Student Services, University of Michigan School of Dentistry, Ann Arbor, MI 48109, USA; 5 Dental Public Health Residency, Texas A & M College of Dentistry, Dallas, TX 75246, USA; 6 Pre-Doctoral Clinical Education, University of Michigan School of Dentistry, Ann Arbor, MI 48109, USA

**Keywords:** mobile dentistry, portable dentistry, legislation, access

## Abstract

The Action for Dental Health Act of 2017 bill is intended to prevent dental disease and divert dental emergencies from high-cost centers (like hospital emergency rooms) to dental offices. Lines 15–17 of the bill include grant funding to support portable or mobile dental equipment, and this should lead to an expansion of opportunities to deliver and receive care through the use of portable dental equipment and mobile dental vans, i.e., portable and mobile dentistry (PMD). Historically, PMD has been valuable to bridge the access gap for those for whom transport can be a challenge, like children and the elderly. However, PMD could be valuable to large employers, allowing the employees to receive dental care with minimal disruption to their workday. Oral pain is known to affect work and school attendance, and improving access to dental care could benefit individuals, families, organizations, and communities.

## 1. Introduction

On 26 February 2018, the United States (US) House of Representatives voted 387–13 in favor of the Action for Dental Health Act of 2017 [1]. Thematically, this bill is intended to prevent dental disease, divert dental emergencies from high-cost centers (like hospital emergency rooms) to dental offices, and reduce barriers to accessing care, including socio-cultural barriers. To truly achieve these lofty goals, innovative strategies are needed for certain populations such as those in long-term care and rural populations lacking access to dental services. Lines 15–17 of the bill include grant funding to support portable or mobile dental equipment which may lead to an expansion of opportunities to deliver and receive care through the use of portable dental equipment and mobile dental vans. Portable dental equipment includes relatively lightweight and easily movable dental chairs, x-ray units, and portable dental drills [2]. Mobile dental units are motorized vehicles that are self-contained dental operatories with dental chairs, x-rays units, and fixed dental drills [3]. In this paper, the term portable and mobile dentistry (PMD) describes the use of portable dental equipment only, mobile dental units only, or their combination. Historically, PMD use had been limited to certain states with large rural populations (like California and Texas) and almost exclusively in the non-profit sector. The current legislation encourages an innovative use of PMD in other environments including the for-profit sector. This paper describes the current state of PMD and highlights successful models that may be replicated and future opportunities the new legislation affords.

## 2. Methods

We reviewed the State Boards of Dentistry websites and evaluated available data related to the current regulation of PMD in the United States. We also reviewed evaluations of the effectiveness of PMD in reaching vulnerable populations. This article is a summary of opportunities and limitations in delivering and receiving care through PMD. This was a literature review, and no IRB approval was required.

## 3. Results

We found that the regulation of PMD can be complicated and highly variable from state to state. Table 1 shows that, across the United States, only 14 states have a specific regulation on mobile dental clinics, and the majority of these require a specific permit (see Table 1) [4]. Additionally, 13 of the 14 states include a specific regulation and guidance about post-operative care. Currently in the US, PMD programs are supported by various private foundations and public financing. The following programs are foundation- and publicly supported. In some states, the variability of regulation may make entry into this field challenging. For example, most states require a mobile clinic permit; however, Arkansas charges $5,000 for this privilege, while California charges only $100 [4]. It should be noted that these fees relate to mobile dental units, and there are no clear rulings related to portable dentistry.

Table 2 provides additional resources for those seeking to further investigate PMD in the United States.

## 4. Discussion

A systematic review on the efficacy of mobile dentistry showed that at-risk populations and those lacking access to traditional dental practices were treated by PMD [5]. This 2014 article suggests that the “future looks promising” for mobile dentistry [5]. PMD is particularly valuable to those for whom transport can be a challenge, like children and the elderly. Children depend on their adult caregiver to understand, value, and finance their oral care. Moreover, children usually require their caregiver to escort them to dental appointments. Research has demonstrated that children with poor oral health are three times more likely to miss school because of dental pain [6]. Additionally, absences caused by pain were associated with poorer school performance, but absences due to routine dental care were not [6]. Through the use of PMD, instead of missing school days due to dental pain or even routine dental care, children may be able to have their dental care while at school. Further, a report of effectiveness of PMD was completed by the State University of New York’s (SUNY) School of Public Health [7]. The following were some of the themes of PMD programs uncovered by the SUNY report:

1. The scope of services provided ranges from preventive services to a full complement of dental treatment services.
2. Programs have grown organically to meet the needs of particular populations or geographic areas for oral health services.
3. The geographic locations may change in response to patient population shifting need.
4. Programs reconcile service availability with the uneven distribution of dental providers in certain geographic areas or for particular populations [7].

Further, the authors emphasize that PMD are also positioned to address the oral health service needs of institutionalized populations including the medically fragile [7].

### 4.1. Pediatric PMD

The St. David’s Foundation Dental Program (SDF) has a fleet of nine mobile dental vans that travel to six school districts to provide free dental care to children in the Central Texas region. In order to tackle a large region, the clinical team is divided into four regional teams that spread out across the three-county area. Each regional team has a pair of vans, and the staff is composed of a supervising dentist, a staff dentist, a hygienist, and four dental assistants. The SDF clinical model has been able to achieve school buy-in because the dental staff work around the school and teacher’s schedule to minimize disruption to the classroom. Children are provided free preventive and restorative care on the vans. Once a school is identified, a screening determines the level of a child’s need. A treatment plan will indicate treatment by the dentist, the hygienist, or both providers. This information helps the data analytics team predict how long the regional team will be at each school and how many schools can complete the Dental Program that school year. The St. David’s Foundation Dental Program is a successful PMD model that has been operating for 18 years and demonstrates the effectiveness of PMD to overcome barriers such as transportation, parents missing work, and children missing school because of dental pain or treatment needs. 

The Program also creates access to care for the underserved population in Central Texas who may not receive care otherwise. 

### 4.2. Geriatric PMD

The US geriatric population (over 60 years) is responsible for 50,881 hospital emergency room (ER) visits per year due to a basic dental complaint (caries, pulpal and periapical disease, gingival or periodontal disease) [8]. It is notable that 63.4% of these ER visits were covered by Medicare (possible because Medicare does not cover routine dental care) [9]. Further, research suggest that in comparison to younger age groups, geriatric patients were more likely to be hospitalized after their dental-related ER visit and more likely to end up in a long-/short-term care facility (rather than return home), which incurs enormous subsequent charges [8]. Therefore, any program that serves as an ER diversion for the elderly is of enormous economic value. An excellent example of such a program is the interdisciplinary Program of All-inclusive Care for Elderly (PACE) in Cambridge (MA) [9]. PACE helps to keep aged residents out of nursing homes and emergency rooms and includes dental services [9]. In this unique program, PMD is utilized to deliver dental services to geriatric patients in a setting that facilitates collaborative care. After their first visit, a multi-disciplinary care plan is developed, and care is delivered by interprofessional teams [9]. PMD affords the opportunity to design (or be incorporated into) highly flexible programs like PACE. When geriatric patients are in short-term and long-term care facilities, their mobility is frequently compromised, and PMD becomes a necessity. 

### 4.3. Future Opportunities

Previous research has shown what an advantage PMD is in eliminating the transport barrier for the elderly and school-attending children [10]. In addition to pediatric and geriatric patients, PMD could also reach the site of a large employer to provide services to the employees (of all ages) with minimal disturbance to their work day. Approximately 1.4 million emergency room visits each year are related to basic dental problems like caries and tooth abscesses [11], and 175,000 hospitalization days per year are associated with a primary diagnosis of a basic dental condition [12]. Simplifying access to care by bringing care to the patient may help to reduce days of lost work due to dental conditions.

Additionally, PMD eliminates the commute time, which may also help patients gain access to dental care. There are benefits to the dentist also: rather than relying on each of 15–20 patients to keep their appointments, the dentist travels to the facility where the patients are already located. This has potential to make work more efficient for the dental team if a failed appointment can be rapidly replaced by another on-site patient.

Larger states like Minnesota with a lot of smaller communities that lack suitable access to a doctor, dentist, or dental therapist may benefit the most from PMD. Small populations may contraindicate setting up a fixed practice and, in contrast, represent an ideal situation for PMD to thrive. PMD affords the opportunity to address this by enabling providers to (perhaps) remain based in their fixed site clinic but travel to underserved areas and provide care as needed. There is also opportunity for thoughtful implementation of PMD programs that are customized to the needs of local populations. For example, a Mandarin-speaking provider may choose to travel to various regions where locals are more comfortable conversing in Mandarin. This may have a powerful impact on dental care access for those communities for whom communication and access were a two-fold barrier to care.

PMD offers many benefits to the patients as well. Being treated on site (at work, school, or other) means minimal interruption to their day. Additionally, dental anxiety may be reduced because, rather than being in a dentist’s office, care is received in an environment that may be more familiar to the patient – there may be less opportunity for “white coat fever” to occur, as patients are away from the traditional dentists’ office. While we recognize that a lifelong relationship with a dentist in a traditional office may be more familiar and comfortable than a PMD setting, the PMD setting may prove more comfortable for some patients. 

While our paper focuses on the value of PMD to improve access to dental services for certain populations, it should also be noted that the discipline of Forensic Odontology has been using PMD as standard armamentarium for decades. Being able to transport dental equipment to a site requiring forensic examination has clear benefits, allowing for a rapid response to the required information for a dental comparison and subsequent identification.

### 4.4. Limitations

The challenge of public health in the US is the independence and authority afforded to each state. This allows each state to deliver regionally customized programs and legislation; however, this limits the ability to establish best practices across the nation. 

Secondly, the regulations in some states may make entry into the PMD field challenging. For example, among states that require a PMD permit, Arkansas charges $5,000 for this permit, while California charges only $100 [4]. It should be noted that these fees are specifically related to mobile dental units, and there are no clear rulings related to portable dentistry. Additionally, some states regulations completely restrict the potential growth of PMD [4]. For example, Louisiana requires pediatric patients to be treated in a private room [4]. Similarly, many states require patients to have access to a toilet, which can limit the possible venues for care delivery [4]. In Texas, a parental accompaniment rule exists, which states that parents must be present in order to bill Medicaid. This policy may be a challenge for PMD programs that travel to schools to provide care. 

Additionally, cost is an important barrier for entry into the market of delivery care through PMD. Although portable dental equipment is relatively inexpensive ($15,000–$20,000), mobile dental vans can cost over $300,000. Further cost challenges include the necessity for potentially expensive support mechanisms to operate efficiently. Support systems include IT and tech/security support services, an experienced driver, many external vendors, and staff to monitor compliance and regulations. To increase the longevity of the mobile van’s life, quarterly maintenances need to be performed by an external dental supply vendor, and proper tying down of the equipment before each van move and optimal, daily maintenance by staff need to be optimal. Some of the other external vendors that are beneficial for mobile dental van programs include an equipment service company, a grey water disposal company and a cleaning crew for deeper, detailed cleanings. Staff that work on mobile dental vans need to be more aware of optimizing space for storage and observe Health Insurance Portability and Accountability Act (HIPAA) regulations within confined spaces. 

Nonetheless, a major challenge to a patient receiving care through PMD is the potential lack of availability for follow-up and management of complications, because the PMD team may be at a different site on the post-treatment day. A PMD site may be established specifically because there are no other dentists available. Hence, partnership with primary care providers and other local clinics would be necessary to manage urgent postoperative matters. Dentists in traditional practices create partnerships and systems to manage their emergencies when they are on vacation, and a similar collaboration with nearby care providers may be plausible here.

## 5. Conclusions

PMD provides many benefits to patients and providers and has been highly effective in serving certain groups such as children and the elderly. Opportunities exist to deliver care through mobile clinics or portable dentistry at the location of a large employer. If a dental team can overcome the barriers of entry into this field, a highly satisfying and efficient work environment can be established, with strong benefits of convenience to the patients they serve. The beneficial impact of PMD on health can be particularly evident in schools serving children, in nursing homes serving the elderly, and in rural and remote communities in which access to care remains a challenge. 

## Figures and Tables

**Table 1:** States having specific regulations for portable dentistry.

State	Mobile Clinic Needs Permit or Special Registration with Board of Dentistry	Post-Op Related Regulation
ALABAMA	No	Yes
ARIZONA	Yes	Yes
ARKANSAS	Yes	Yes
CALIFORNIA	Yes	Yes
INDIANA	Yes	Yes
KANSAS	Yes	Yes
LOUISIANA	Yes	Yes
MASSACHUSETTS	Yes	Yes
MISSISSIPPI	Yes	Yes
SOUTH CAROLINA	Yes	Yes
TEXAS	Yes	Yes
VIRGINIA	Yes	Yes
WEST VIRGINIA	Yes	Yes
WISCONSIN	Yes	Yes

All other states not mentioned do not have specific regulations; Adapted from Mobile Health homepage. Accessed 7/19/18 and available at http://adi-mobilehealth.com/wp-content/uploads/2017/01/Mobile-Health-Clinic-Regulations-Federal-State.pdf.

**Table 2:** Additional resources for those interested in conducting more research on mobile dental clinics and portable dentistry.

Topic	Reference and Links
Manual on planning, implementing, and measuring the effectiveness of mobile and portable dental programs.	https://www.mobile-portabledentalmanual.com/
Mobile and portable dental services in Preschool and School settings. Complex issues.	https://www.astdd.org/docs/mobile-portable-astdd-issue-brief-final-02-29-2011.pdf
Association of State and Territorial Dental Directors support school-based mobile and portable dental programs	https://www.astdd.org/docs/school-based-or-school-linked-mobile-or-portable-dental-services-policy-statement-february-28-2012.pdf
An Assessment of mobile and portable dentistry programs to improve population oral health	http://www.chwsny.org/wp-content/uploads/2017/09/OHWRC_Mobile_and_Portable_Dentistry_Programs_2017.pdf
Mobile dental clinic state and federal laws and regulations	http://adi-mobilehealth.com/wp-content/uploads/2017/01/Mobile-Health-Clinic-Regulations-Federal-State.pdf
